# Children's Hospital, Nottingham

**Published:** 1899-02-11

**Authors:** Catherine A. Morrish


The following statement accompanies the above letter:—

STATEMENT BY CATHERINE A. MORRISH, IN ANSWER TO THE COMMITTEE OF THE NOTTINGHAM HOSPITAL FOR CHILDREN.

I affirm the substantial truth of my former statement. I am not conscious that it contains any inaccuracy introduced by inadvertence. I positively declare that I had never given an enema until I did so in the isolation ward. When I did this nobody was present to direct me. Up to this time I had not received any instruction in the use of the thermometer. When I went to nurse in the isolation block I was shown how to use the thermometer; that was the first chart that I had ever marked. I declare that the latest time at night that I was ever visited, when on isolation duty, was about ten o'clock. On one occasion only the matron called about that hour. I declare that the only table, off which I had to eat my food, was the general table in the ward in which the patient was; the table in the little room was merely a dressing-table or washstand. I declare that the only conveniences for washing were the doctor's basin, which has his towel passed through the handle, and the sink in the scullery underneath the uncurtained window. I know of no other item of my statement to which the management have taken exception.

February 6th, 1899. CATHERINE A. MORRISH.

